# Supplementary material for: Pyruvate Dehydrogenase Kinase 1 inhibition mediated oxidative phosphorylation enhancement in cartilage promotes osteoarthritis progression
Source: BMC Musculoskelet Disord. 2023 Jul 20;24:597. doi: 10.1186/s12891-023-06585-6 (PMC10357736; doi:10.1186/s12891-023-06585-6)
Supplement: Supplementary file 1 — Additional file 1. [file 12891_2023_6585_MOESM1_ESM.zip › KEGG.Gsea.1653622432464/KEGG_PYRUVATE_METABOLISM.html]

Details for gene set KEGG\_PYRUVATE\_METABOLISM[GSEA]

|  || Dataset | 55457\_collapsed\_to\_symbols.cls.cls #OA\_versus\_normal.cls.cls #OA\_versus\_normal\_repos |
| Phenotype | cls.cls#OA\_versus\_normal\_repos |
| Upregulated in class | OA |
| GeneSet | KEGG\_PYRUVATE\_METABOLISM |
| Enrichment Score (ES) | 0.5206598 |
| Normalized Enrichment Score (NES) | 1.953494 |
| Nominal p-value | 0.0023201855 |
| FDR q-value | 0.0038274408 |
| FWER p-Value | 0.028 |
Table: GSEA Results Summary

  

Fig 1: Enrichment plot: KEGG\_PYRUVATE\_METABOLISM      
 Profile of the Running ES Score & Positions of GeneSet Members on the Rank Ordered List

  

| SYMBOL | TITLE | RANK IN GENE LIST | RANK METRIC SCORE | RUNNING ES | CORE ENRICHMENT || 1 | PDHB | pyruvate dehydrogenase E1 subunit beta [Source:HGNC Symbol;Acc:HGNC:8808] | 149 | 0.862 | 0.0608 | Yes |
| 2 | MDH1 | malate dehydrogenase 1 [Source:HGNC Symbol;Acc:HGNC:6970] | 271 | 0.772 | 0.1163 | Yes |
| 3 | GRHPR | glyoxylate and hydroxypyruvate reductase [Source:HGNC Symbol;Acc:HGNC:4570] | 363 | 0.720 | 0.1697 | Yes |
| 4 | DLD | dihydrolipoamide dehydrogenase [Source:HGNC Symbol;Acc:HGNC:2898] | 565 | 0.635 | 0.2074 | Yes |
| 5 | MDH2 | malate dehydrogenase 2 [Source:HGNC Symbol;Acc:HGNC:6971] | 615 | 0.618 | 0.2554 | Yes |
| 6 | PCK2 | "phosphoenolpyruvate carboxykinase 2, mitochondrial [Source:HGNC Symbol;Acc:HGNC:8725]" | 759 | 0.579 | 0.2929 | Yes |
| 7 | ALDH2 | aldehyde dehydrogenase 2 family member [Source:HGNC Symbol;Acc:HGNC:404] | 1003 | 0.517 | 0.3174 | Yes |
| 8 | ACAT1 | acetyl-CoA acetyltransferase 1 [Source:HGNC Symbol;Acc:HGNC:93] | 1017 | 0.513 | 0.3596 | Yes |
| 9 | ALDH3A2 | aldehyde dehydrogenase 3 family member A2 [Source:HGNC Symbol;Acc:HGNC:403] | 1263 | 0.465 | 0.3796 | Yes |
| 10 | HAGH | hydroxyacylglutathione hydrolase [Source:HGNC Symbol;Acc:HGNC:4805] | 1395 | 0.443 | 0.4066 | Yes |
| 11 | ME1 | malic enzyme 1 [Source:HGNC Symbol;Acc:HGNC:6983] | 1746 | 0.392 | 0.4124 | Yes |
| 12 | ME2 | malic enzyme 2 [Source:HGNC Symbol;Acc:HGNC:6984] | 1870 | 0.377 | 0.4344 | Yes |
| 13 | GLO1 | glyoxalase I [Source:HGNC Symbol;Acc:HGNC:4323] | 2141 | 0.345 | 0.4425 | Yes |
| 14 | ALDH7A1 | aldehyde dehydrogenase 7 family member A1 [Source:HGNC Symbol;Acc:HGNC:877] | 2158 | 0.343 | 0.4700 | Yes |
| 15 | ACYP2 | acylphosphatase 2 [Source:HGNC Symbol;Acc:HGNC:180] | 2346 | 0.321 | 0.4825 | Yes |
| 16 | PDHA1 | pyruvate dehydrogenase E1 subunit alpha 1 [Source:HGNC Symbol;Acc:HGNC:8806] | 2433 | 0.310 | 0.5018 | Yes |
| 17 | PC | pyruvate carboxylase [Source:HGNC Symbol;Acc:HGNC:8636] | 2629 | 0.287 | 0.5108 | Yes |
| 18 | ALDH9A1 | aldehyde dehydrogenase 9 family member A1 [Source:HGNC Symbol;Acc:HGNC:412] | 2795 | 0.270 | 0.5207 | Yes |
| 19 | AKR1B1 | aldo-keto reductase family 1 member B [Source:HGNC Symbol;Acc:HGNC:381] | 3138 | 0.236 | 0.5139 | No |
| 20 | PKM | pyruvate kinase M1/2 [Source:HGNC Symbol;Acc:HGNC:9021] | 3963 | 0.159 | 0.4633 | No |
| 21 | ME3 | malic enzyme 3 [Source:HGNC Symbol;Acc:HGNC:6985] | 4613 | 0.104 | 0.4215 | No |
| 22 | LDHAL6B | lactate dehydrogenase A like 6B [Source:HGNC Symbol;Acc:HGNC:21481] | 5040 | 0.070 | 0.3943 | No |
| 23 | DLAT | dihydrolipoamide S-acetyltransferase [Source:HGNC Symbol;Acc:HGNC:2896] | 5647 | 0.020 | 0.3489 | No |
| 24 | ACAT2 | acetyl-CoA acetyltransferase 2 [Source:HGNC Symbol;Acc:HGNC:94] | 6291 | -0.027 | 0.3011 | No |
| 25 | ALDH1B1 | aldehyde dehydrogenase 1 family member B1 [Source:HGNC Symbol;Acc:HGNC:407] | 6551 | -0.046 | 0.2848 | No |
| 26 | LDHC | lactate dehydrogenase C [Source:HGNC Symbol;Acc:HGNC:6544] | 7603 | -0.127 | 0.2138 | No |
| 27 | LDHB | lactate dehydrogenase B [Source:HGNC Symbol;Acc:HGNC:6541] | 7718 | -0.135 | 0.2163 | No |
| 28 | PCK1 | phosphoenolpyruvate carboxykinase 1 [Source:HGNC Symbol;Acc:HGNC:8724] | 9100 | -0.239 | 0.1291 | No |
| 29 | PDHA2 | pyruvate dehydrogenase E1 subunit alpha 2 [Source:HGNC Symbol;Acc:HGNC:8807] | 9124 | -0.241 | 0.1476 | No |
| 30 | ACACA | acetyl-CoA carboxylase alpha [Source:HGNC Symbol;Acc:HGNC:84] | 9177 | -0.246 | 0.1642 | No |
| 31 | ACYP1 | acylphosphatase 1 [Source:HGNC Symbol;Acc:HGNC:179] | 9574 | -0.280 | 0.1570 | No |
| 32 | PKLR | pyruvate kinase L/R [Source:HGNC Symbol;Acc:HGNC:9020] | 10120 | -0.333 | 0.1426 | No |
| 33 | ACACB | acetyl-CoA carboxylase beta [Source:HGNC Symbol;Acc:HGNC:85] | 10955 | -0.425 | 0.1135 | No |
| 34 | LDHA | lactate dehydrogenase A [Source:HGNC Symbol;Acc:HGNC:6535] | 11134 | -0.448 | 0.1373 | No |
Table: GSEA details [plain text format]

  

Fig 2: KEGG\_PYRUVATE\_METABOLISM      
 Blue-Pink O' Gram in the Space of the Analyzed GeneSet

  

Fig 3: KEGG\_PYRUVATE\_METABOLISM: Random ES distribution      
 Gene set null distribution of ES for **KEGG\_PYRUVATE\_METABOLISM**
